# Cancer Stem-Like Cells Enriched in Panc-1 Spheres Possess Increased Migration Ability and Resistance to Gemcitabine

**DOI:** 10.3390/ijms12031595

**Published:** 2011-03-01

**Authors:** Tao Yin, Hongji Wei, Shanmiao Gou, Pengfei Shi, Zhiyong Yang, Gang Zhao, Chunyou Wang

**Affiliations:** 1 Pancreatic Center, Department of General Surgery, Union Hospital, Tongji Medical College, Huazhong University of Science and Technology, Wuhan, Hubei Province, 430022, China; E-Mails: whj86027@smail.hust.edu.cn (H.W.); gousm@163.com (S.G.); fox-shi@foxmail.com (P.S.); dryzy@163.com (Z.Y.); zhaogang1427@yahoo.com.cn (G.Z.); 2 Hubei Key Laboratory of Biological Targeted Therapy, Wuhan, Hubei Province, 430022, China

**Keywords:** pancreatic cancer, Bmi-1, chemoresistance, invasion, tumorigenesis

## Abstract

Pancreatic cancer is one of the most lethal malignancies with poor prognosis. Previously, we found that a subpopulation of cancer stem cells (CSCs) in the Panc-1 pancreatic cancer cell line could propagate to form spheres. Here we characterized the malignant phenotypes of the pancreatic cancer stem CD44+/CD24+ cells, which were enriched under sphere forming conditions as analyzed by flow cytometry. These cells demonstrated increased resistance to gemcitabine and increased migration ability. Moreover, these cells exhibited epithelial to mesenchymal transition characterized by a decreased level of the epithelial marker E-cadherin and an increased level of the mesenchymal marker vimentin. Notably, abnormal expression of Bmi-1, ABCG2, Cyclin D1 and p16 were found in Panc-1 CSCs. Our results suggest that targeted inhibition of CSCs represents a novel therapeutic approach to overcome chemoresistance and metastasis of pancreatic cancer.

## 1. Introduction

Pancreatic cancer is currently the fourth leading cause of cancer-related mortality in Western countries. Despite advances in tumor biology and the development of cancer therapeutic strategies, the prognosis of pancreatic cancers remains miserable, with the five year survival rate less than 5% [1]. The poor prognosis of pancreatic cancer is mainly attributed to the malignant behavior of pancreatic cancer. Extensive local invasion and early systemic dissemination are hallmarks of pancreatic cancer. Moreover, pancreatic cancer is resistant to most cytotoxic drugs. The high recurrence rate after operation remains a big problem for clinicians.

The discovery of cancer stem cells (CSCs) has changed our view of carcinogenesis and cancer therapy. It is proposed that subgroups of cancer stem cells within the tumor sustain the formation and growth of the tumor and account for tumor recurrence due to their self-renewal and differentiation abilities, metastatic potential, and resistance to conventional therapeutics [2–4]. Cancer stem cells have been identified and isolated from a variety of cancers including the blood, breast and central nervous system and pancreas cancers [5–9]. Thus, further characterization of pancreatic cancer stem cells may improve the prognosis and therapy of pancreatic cancer.

In a previous study, we found that a subpopulation of cells in the Panc-1 pancreatic cancer cell line could propagate to form spheres, and strikingly these cells exhibit features of CSCs such as extensive self-renewal, proliferation, differentiation, and tumorigenesis [10]. On this basis, we went further to characterize the malignant phenotypes of these pancreatic cancer stem cells such as chemoresistance and motility and investigate the potential mechanisms responsible for these phenotypes.

## 2. Results and Discussion

### 2.1. CSC Population of Panc-1 Cells Is Enriched under Sphere-Forming Conditions

**Figure 1.**
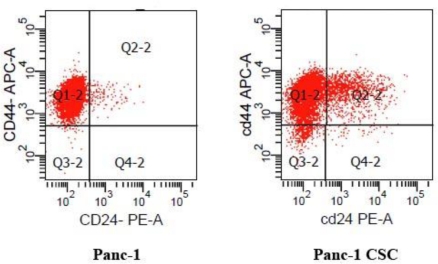
The Panc-1 cell cancer stem cell (CSC) subpopulation (CD44+/CD24+) was enriched under sphere-forming conditions. The right upper quadrant (Q2-2) indicates the distribution of the CD44+/CD24+ subgroup of cells as analyzed by flow cytometry. The experiment was repeated 5 times and the data are expressed as mean ± standard deviation, statistically significant differences were determined by Student’s t test, the difference between the Panc-1 group and the Panc-1 CSC group was significant, p < 0.05.

Cell surface markers have been widely used to isolate stem cells. The surface markers CD44, CD24, and ESA have been well defined for isolating CSCs from primary pancreatic adenocarcinomas [9]. The CD44+/CD24+ subpopulation isolated from Panc-1 cells was considered to consist of pancreatic cancer cells with stem cell properties due to their increased *in vitro* clonogenic and *in vivo* tumor initiating potential [11]. By flow cytometry analysis, we found that the CD44+/CD24+ subpopulation of cells accounted for 12.6 ± 5.10% in the Panc-1 CSC group but only accounted for 1.43 ± 0.59% in Panc-1 cells (Figure 1). These results indicated that the pancreatic cancer cells with stem cell properties were enriched under sphere forming conditions.

### 2.2. Panc-1 CSCs Exhibit Increased Chemoresistance to Gemcitabine

**Figure 2.**
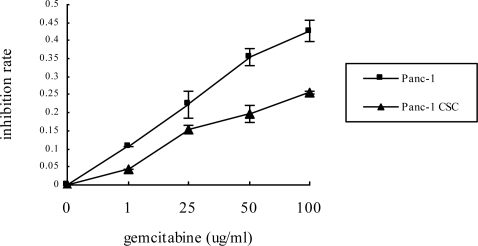
Panc-1 CSCs have increased chemoresistance to gemcitabine. Panc-1 CSCs or Panc-1 cells were treated with various dosages of gemcitabine for 48 h. Cell viability was determined by MTT assay. Panc-1 CSCs showed increased resistance to gemcitabine compared to Panc-1 cells cultured in monolayer.

As the first step to characterize the phenotypes of the pancreatic CSCs, we sought to determine their chemosensitivity. Both Panc-1 cells and Panc-1 CSC were exposed to the most widely used chemotherapeutic agent: gemcitabine. The cell viability was determined by MTT assay and the results demonstrated that while Gemcitabine inhibited the proliferation of Panc-1 cells and Panc-1 CSC in a dose dependent manner, Panc-1 CSCs showed a relative resistance to gemcitabine compared to Panc-1 cells cultured in monolayer, and the difference between the growth inhibition rate of these two groups was statistically significant (P < 0.05) (Figure 2). These data demonstrate that Panc-1 CSCs exhibit increased resistance to gemcitabine compared to Panc-1 cancer cells.

### 2.3. Panc-1 CSCs Exhibit Increased Migration Ability

**Figure 3.**
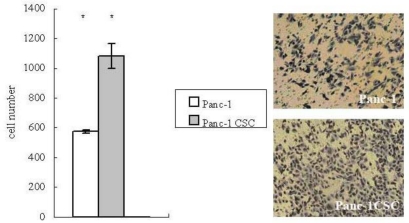
Panc-1 CSCs have increased migration ability. The migration of Panc-1 CSCs or Panc-1 cells was determined by transwell migration assay. After the filter was fixed and stained, the number of cells migrated to the undersurface of the filter was compared. Left side: Panc-1 CSCs had increased migration ability compared to Panc-1 cells, (40× fold). * p < 0.05. Right side: images of Panc-1 cells (top) and Panc-1 CSCs (bottom).

Recent evidence suggests that CSCs may drive the progression and metastasis of malignant cancer [12]. Since metastasis depends on increased motility of cancer cells, we examined the migration ability of Panc-1 CSCs by transwell migration assay. It was observed that larger numbers of Panc-1 CSCs migrated to the lower side of the membrane compared to Panc-1 cells (Figure 3), suggesting that Panc-1 CSCs have increased migration ability.

### 2.4. Panc-1 CSCs Exhibit Epithelial to Mesenchymal Transition (EMT)

**Figure 4.**
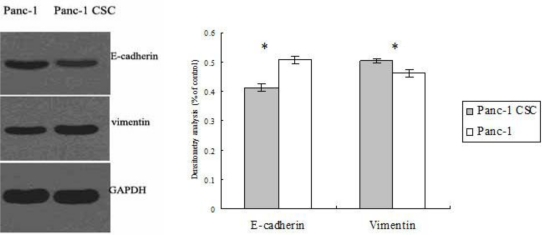
Panc-1 CSCs demonstrate a phenotype suggestive of epithelial to mesenchymal transition (EMT). The expression levels of E-cadherin and vimentin in Panc-1 CSCs and Panc-1 cells were determined by Western blot analysis (left). Densitometry analysis revealed the differences between Panc-1 CSCs and Panc-1 cells (right). * p < 0.05.

Tumor metastasis is often correlated with loss of epithelial properties and acquisition of the fibroblast-like phenotype of cancer cells, a phenomenon known as EMT [13]. Thus, we further investigated whether the increased motility of Panc-1 CSCs results from EMT. By Western blot analysis we found that the protein level of the epithelial marker E-cadherin was decreased, and that of the mesenchymal marker vimentin increased in Panc-1 CSCs compared with Panc-1 cancer cells (Figure 4), indicating that Panc-1 CSC had undergone EMT.

### 2.5. Abnormal Expression of Bmi-1, ABCG2, Cyclin D1 and p16 in Panc-1 CSCs

**Figure 5.**
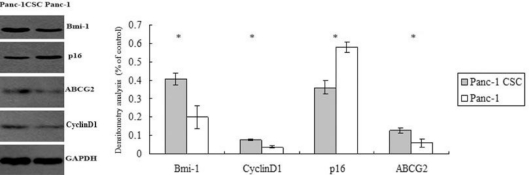
Abnormal expression of Bmi-1, ABCG2, Cyclin D1 and p16 in Panc-1 CSCs. The levels of Bmi-1, ABCG2 and p16 in Panc-1 CSCs and Panc-1 cells were determined by Western blot analysis and normalized to GAPDH (left). Densitometry analysis revealed the differences between Panc-1 CSCs and Panc-1 cells. * p < 0.05.

We further explored the possible mechanisms responsible for the malignant phenotypes of Panc-1 CSCs as shown above. Multidrug resistance is an important mechanism for pancreatic cancer to acquire chemoresistance and ATP-binding cassette (ABC) transporters participate in pumping the drug out of the cancer cells and endowing multidrug resistance. By Western blot analysis, we found that ABCG2/BCRP1 was overexpressed in Panc-1 CSCs compared to Panc-1 cells. In addition, Cyclin D1, an important regulator of cell cycle progression and proliferation, was overexpressed in Panc-1 CSCs. p16, one of the important regulators of cellular senescence and apoptosis, was downregulated in Panc-1 CSCs. Bmi-1, which is an oncogene and stem cell renewal factor, was also highly expressed in Panc-1 CSCs (Figure 5).

### 2.6. Discussion

The cancer stem cell theory posits that a small population of progenitors with extensive self-renewal properties determines the initiation and behavior of tumors. Mounting evidence has demonstrated the important role that cancer stem cells play in tumor initiation, maintenance, progression and recurrence [14]. Moreover, cancer stem cells display increased resistance to apoptosis induced by cytotoxic agents and radiation therapy [15]. The intrinsic resistance to apoptosis may endow CSCs with chemoresistant ability. In the present study, we found that Panc-1 CSCs showed increased resistance to commonly used chemotherapeutic drugs compared to Panc-1 cells. These results validate the point that current therapies might target the bulk of the tumor, but the resistant cancer stem cells may be enriched and rapidly repopulate the tumor from which they originated after chemotherapy [16]. As for pancreatic cancer, the resistant cancer stem cells may be the origin of recurrence after chemotherapy and measures to eradicate these cells may be crucial for improving the prognosis of pancreatic cancer.

We further explored the possible mechanisms by which the resistance to chemotherapy may be regulated in Panc-1 CSCs. The epigenetic regulator Bmi-1 belongs to the polycomb group family, which plays essential roles in the self-renewal and propagation of normal and cancer stem cells [17]. In many cases, Bmi-1 functions as an oncogene and endows the cancer cells with strong antiapoptotic properties via dysregulation of multiple mechanisms [18]. Moreover, Bmi-1 is up-regulated in pancreatic cancer and this is related to the poor prognosis of pancreatic cancer patients [19]. In our study, Bmi-1 is expressed at high levels in Panc-1 CSCs compared to Panc-1 cells cultured in monolayer. This indicated that Bmi-1 may not only contribute to maintain the cancer stem cells in pancreatic cancer, but will also endow abnormal growth and chemoresistant ability to the CSCs.

Interestingly, we found that the expression of the ABC transporter ABCG2 was increased in Panc-1 CSCs compared to Panc-1 cells cultured in monolayer. ABCG2 belongs to the ABC transporter family, which protects cells by pumping a variety of cytotoxic agents out of the cells. This property endows the multidrug resistance to cancer cells and is a major obstacle to successful cancer chemotherapy [20]. Moreover, the drug-transporting property conferred by the ABCG2 transporter results in diminished intracellular accumulation of Hoechst 33342, accounting for the increased side population (SP) phenotype. Indeed, increased ABCG2 expression may be a common feature of pluripotent stem cells [21]. In our study, increased expression of ABCG2 further verifies the stem cell properties of Panc-1 CSCs.

Our results also demonstrate that p16, a regulator of cell senescence and cell cycle, was downregulated in Panc-1 CSCs. Loss of p16 function may be involved in the carcinogenesis and aggressive behavior of pancreatic cancer [22]. The downregulation of p16 in Panc-1 CSCs may explain the high propagation capacity of Panc-1 spheres cultured both *in vitro* and *in vivo* as demonstrated in our previous study [10]. p16 is also implicated in the apoptosis and chemotherapy of pancreatic cancer. In many cases, p16 expression has been used to determine the sensitivity of cancer cells to chemotherapy and adenoviral-mediated p16INK4A reintroduction could greatly enhance the cytotoxicity of 5-Fluorouracil or gemcitabine in Panc-1 pancreatic adenocarcinoma cells [23]. In our study, the downregulation of p16 in Panc-1 CSC may partly account for the resistance of Panc-1 CSCs to chemotherapy.

Cyclin D1 is an important regulator of cell cycle progression. The overexpression of cyclin D1 has been linked to the development and progression of pancreatic cancer through promoting abnormal growth and resistance to chemotherapy [24,25]. Moreover, recently studies have revealed that cyclin D1 kinase activity is required for the self-renewal of mammary stem and progenitor cells [26]. Therefore, our results indicate that high expression of cyclin D1 may help maintain and regulate the biology of cancer stem cells in Panc-1 cells and multiple mechanisms cooperate to contribute to the resistance of Pan-1 CSCs to chemotherapy.

Recent evidence has supported the link of chemoresistance and EMT with stem cell phenotype [27]. EMT is known to promote the invasion and metastasis of tumors [28]. However, emerging lines of evidence suggest molecular and phenotypic associations between apoptotic resistance and the acquisition of an EMT–like phenotype in cancer cells [29,30]. In our experiments, Panc-1 CSCs exhibited increased migration ability compared to Panc-1 cells and demonstrated downregulation of the epithelial marker E-cadherin and upregulation of the mesenchymal marker vimentin. Thus, we conclude that Panc-1 CSCs undergo EMT. Considering the role EMT plays in invasiveness and dissemination of cancer, such features may facilitate the escape of the resistant Panc-1 CSCs during chemotherapy.

## 3. Experimental Section

### 3.1. Cell Lines and Cell Culture

The pancreatic cancer cell line Panc-1 originated from ATCC (Manassas, VA, USA), and was cultured in DMEM supplemented with 10% fetal bovine serum, 100 U/mL pencillin and 100 U/mL streptomycin at 37 °C with 5% CO_2_. The culture conditions for Panc-1 cells to form tumor spheres in suspension was as described previously [10]. The sphere formation media (SFM) used was DMEM-F12 supplemented with 10 ng/mL fibroblast growth factor-basic (Peprotech), 20 ng/mL epidermal growth factor (Peprotech), 5 μg/mL insulin, 2.75 μg/mL transferrin, 2.5 ng/mL sodium selenite (Sigma), and 0.4% bovine serum albumin (Amresco). Briefly, the enzymatically dissociated single cells were diluted to a density of 10^3^ cells/mL with SFM, and plated into the low attached plate. The cells were passaged every 10 to 14 days and replated in the SFM. The spherical clusters of cells grown under these conditions were named Panc-1 CSCs.

### 3.2. Flow Cytometry Assay

Panc-1 cells were dissociated with trypsin-EDTA solution (trypsin, 0.25%; EDTA, 0.02%) and washed twice with phosphate-buffered saline (PBS) and resuspended in PBS at a density of 1 × 10^6^ cells/100 μL. The dissolved cells were stained using APC antihuman CD44, PE anti-human CD24 and ESA-FITC (Biolegend) at a dilution of 1:40, incubated for 20 min on ice and then washed twice with PBS. The respective isotype controls were used at concentrations according to the manufacturer’s instruction. The samples were analyzed on a flow cytometer (BD LSR II, America) and the data were analyzed with the BD FACS Diva software.

### 3.3. MTT Cell Proliferation Assay

Three groups were divided: blank group (no cells), control group (no treatment), experiment group (gemcitabine treatment). Cells were plated in 96 well plates with a density of 10,000 cells/well. 24 h later the cells were incubated with medium containing various concentration of gemcitabine (Eli Lilly) for 48 h. Then 5 mg/mL of 3-(4,5-dim ethylthiazol-2-yl)-2,5-diphenyl-2H-tetrazolium bromide (MTT) (Sigma) was added to each well. After incubation for 4 h, the supernatant was replaced with 150 μL of dimethyl sulfoxide (Sigma). The absorption (A) was read at 490 nm using a spectrophotometer. The inhibition rate of cell proliferation was calculated as: (Acontrol − Ablank) − (AexperimentAblank)/(Acontrol − Ablank) × 100%. The experiments were performed in triplicate and repeated twice.

### 3.4. Transwell Migration Assay

The migration assay was performed using transwell cell culture chambers (8 μM pore size polycarbonate membrane, Costar). Briefly, cells were resuspended in DMEM with 1% FBS to a concentration of 5 × 10^5^/mL. The upper chamber was loaded with 100 μL of cell suspension and the lower chamber was loaded with 600 μL of DMEM with 20% FBS. After incubation for 24 h at 37 °C with 5% CO_2_, the filter was fixed with 4% paraformaldehyde and stained with hemaroxylin. The cells on the upper side of the filter were wiped off with a cotton swab. The cells migrated to the undersurface of the membrane were counted under microscope. Ten microscopic fields (400×) were randomly selected to count cells. Each assay was done in triplicate.

### 3.5. Western Blot Analysis

Pancreatic cancer cells were lysed in lysis buffer (20 mM Tris pH 7.5, 150 mM NaCl, 1% Triton X-100, 0.1% NP40, 0.5% sodium deoxycholate, 1 mM of phenylmethylsulfonyl fluoride and gabexate mesilate). The protein concentration of the lysate was quantitated by BSA method. Equal amounts of lysate were loaded and separated by SDS-polyacrylamide gels, and transferred onto nitrocellulose membranes. The membranes were blocked with 5% non-fat milk powder in TBS for 1 h and probed with primary antibodies against vimentin (Sigma, 1:200 dilution), E-cadherin (Santa Cruz, 1:500 dilution), p16 (Santa Cruz, 1:400 dilution), ABCG2 (Santa Cruz, 1:300 dilution) or GAPDH (ProMab, 1:8000 dilution). After washing with TBS-T, the membrane was incubated with secondary horseradish peroxidase-coupled antibodies and visualized using enhanced chemiluminescence.

### 3.6. Statistical Analysis

The differences between different groups were evaluated using the Student’s t test. P < 0.05 was considered significant.

## 4. Conclusions

In summary, our results indicate that cancer stem cells are enriched in Panc-1 spheres and they demonstrate an EMT phenotype and increased resistance to gemcitabine and motility. Overexpression of Bmi-1, ABCG2 and cyclin D1 and downregulation of p16 could contribute to these phenotypic changes of Panc-1 cancer stem cells.
